# Comprehensiveness of online sources for patient education on hereditary hearing impairment

**DOI:** 10.3389/fped.2023.1147207

**Published:** 2023-06-19

**Authors:** Yaşar Kemal Duymaz, Ahmet M. Tekin, Patrick D’Haese, Şamil Şahin, Burak Erkmen, Ahmet Adnan Cırık, Vedat Topsakal

**Affiliations:** ^1^ Department of Otolaryngology, University of Health Science, Umraniye Training and Research Hospital, Istanbul, Türkiye; ^2^ Department of Otolaryngology and Head & Neck Surgery, Vrije Universiteit Brussel, University Hospital UZ Brussel, Brussels Health Campus, Brussels, Belgium; ^3^ Faculty of Medicine and Pharmacy, Vrije Universiteit Brussel, Brussels, Belgium; ^4^ Private ENT Practice, Istanbul, Türkiye; ^5^ Department of Otolaryngology, University of Health Science, Sancaktepe Prof Dr Ilhan Varank Training and Research Hospital, Istanbul, Türkiye

**Keywords:** sensorineural hearing loss, hereditary hearing impairment, genetic deafness, hereditary hearing loss, patient education material, readability assessment

## Abstract

**Introduction:**

The present study aimed at investigating the readability of online sources on hereditary hearing impairment (HHI).

**Methods:**

In August 2022, the search terms “hereditary hearing impairment”, “genetic deafness”, hereditary hearing loss”, and “sensorineural hearing loss of genetic origin” were entered into the Google search engine and educational materials were determined. The first 50 websites were determined for each search. The double hits were removed and websites with only graphics or tables were excluded. Websites were categorized into either a professional society, a clinical practice or a general health information website. The readability tests to evaluate the websites included: Flesch Reading Ease, Flesch–Kincaid grade level, Gunning–Fog Index, Simple Measure of Gobbledygook, Coleman–Liau Index, Automated Readability Index.

**Results:**

Twentynine websites were included and categorized as from 4 professional societies, 11 from clinical practices and 14 providing general information. All analyzed websites required higher reading levels than sixth grade. On average 12–16 years of education is required to read and understand the websites focused on HHI. Although general health information websites have better readability, the difference was not statistically significant.

**Discussion:**

The readability scores of every type of online educational materials on HHI are above the recommended level indicating that not all patients and parents can comprehend the information they seek for on these websites.

## 1. Introduction

One to three out of thousand children born with a severe to profound sensorineural hearing loss and at least 50% is attributed to a genetic cause (1, 2). There are many research centers focusing on identifying new deafness-causing genes and therefore there is a relative increase in the percentage of hearing losses attributed to genetics (3). As neonatal care improves, there is also a relative decrease in acquired deafness due to neonatal complications (4). Untreated severe (≥90 dB HL) sensorineural hearing loss (SNHL) will endanger timely speech and language development. Early diagnosis and intervention are of vital importance and providing the correct information to patients and parents is crucial (5).

The use of the internet as an information source for health-related issues is gradually increasing (6). In order to achieve positive health outcomes, individuals must be aware of how to take preventive measures against diseases and use available health care resources effectively (7) Patients and parents often consult the internet for information about their health status and treatment (8). It is the second source of health information after the clinician (9). The rate of admission to the internet is increasing particularly in stigmatized illnesses (10). Unfortunately hearing impairment (HI) may be typically accepted as a stigmatized and chronic disease although its often treatable (11). Therefore, the likelihood of parents of children with HI searching the internet is high. Also, the ongoing digitalization and improving access to internet sources will pave the way for more common disease descriptions on the web. The covid-19 pandemic has accelerated this trend and more physicians use internet sources to inform their patients, to collect information with questionnaires prior to the consultation and for teleconsultation (12). All healthcare workers in the field of SNHL know that a cochlear implant is necessary for hearing rehabilitation in patients who do not benefit from conventional hearing devices. Consequently, the educational materials on the internet related to early childhood SNHL become more important. It is hoped that high quality information on the internet accessed by parents of children with SNHL will result in earlier diagnosis and early intervention, leading to more successful rehabilitation. The cause seems very evident, but the quality of the means determines the success.

What defines the quality of an internet source for a patient and for parent education? Readability of the provided text is probably the most determining factor for quality. Thus, the literacy rates of the target population should be considered. Different approaches and methods have been suggested in the literature to determine readability of a text or a website. In the American academic system, the readability of a text is the numerical value corresponding to the academic score required to understand the text. An average American adult reads at an 8th-grade level. The American Medical Association (AMA) and the National Institute of Health recommend writing educational materials at the 6th-grade level (13–15). Insufficient knowledge about the disease or treatment negatively influences health and treatment outcomes (16). However, the websites which parents consult about the medical problems of their children are usually at a reading level that exceeds their reading skills (17).

A set of readability assessment tools are available, each with their own advantages and shortcomings. The literature indicates that 6 tools are popular in otorhinolaryngology to asses readability (18–24). The Flesch Reading Ease (FRE) was created in 1948 and is still widely used (25). It is typically used for the evaluation of medical reading materials written for adults (26, 27). The Flesch–Kincaid Grade Level (FKGL) is used to evaluate academic materials and may be readily translated to the grade of reading level in the United States. It is still used for document standardization by the US military and many institutions (28). The Gunning–Fog Index (GFI) uses the polysyllabic word count and average sentence length in a selected 100-word quotation to determine the readability level. The same variables are used by The Simple Measure of Gobbledygook (SMOG), but in this method the whole text is evaluated. SMOG was advised by the National Cancer Institute for the evaluation of health information (29). SMOG was developed in 1969, and validation tests have demonstrated that it has a significant association with needed text reading levels (27). The Coleman–Liau Index (CLI) formula determines the score level of a written document based on sentence length and number of characters (30). The Automatic Readability Index (ARI) is calculated by using the number of characters, the number of words, and the number of sentences (28).

Readability studies in otology have been published in topics like cochlear implants, tinnitus, and vestibular disorders. However, the area of hereditary hearing impairment (HHI) has not been addressed hitherto which is perhaps even re importance for families with a genetic trait. Moreover, there is a significant underserved population indicated for hearing aids and cochlear implants. As many as 30% of elderly people who could benefit from hearing aids do not have access to them. Similarly, fewer than 10% of profoundly deaf adults have access to a cochlear implant (31, 32). Therefore, it seems evident that parents who need to decide sometimes for a surgical intervention for the placement of a cochlear implant should be informed correctly and adequately, also on the topic of inheritance risks.

The number of children with childhood deafness as a result of hereditary hearing loss are increasing. The internet is a key source of health care information. The readability of health care materials related to HHI and related treatments should be payed attention to. Unfortunately websites providing information on HHI too often use medical jargon. Since there are many complicated issues to be addressed such as heritability, degree of hearing loss and highly technological treatment options such as hearing aids and cochlear implants, most parents at a reading level of AMA 8th grade may not be able to comprehend the information on these websites. In the worst case they may even refrain from seeking help therefore speech and language delay may follow. In the worst case they therefore may even refrain from seeking help, not knowing about the potential speech and language delay that may follow.

Our aims were therefore to study the readability of online sources on HHI: either professional clinical websites or general public websites.

## 2. Materials and methods

In August 2022, Patient Educational Materials (PEM) about “hereditary hearing impairment” were determined by searching in Google Search. The search terms used were “hereditary hearing impairment”, “genetic deafness”, “hereditary hearing loss”, and “sensorineural hearing loss of genetic origin”. In advanced search, only “full sentence” and “English language” were selected. The first 50 websites for each term research were included. A total of 200 websites were evaluated. The double hits, academic journals, videos, and websites that include only graphics or tables were excluded. Websites were divided into 3 categories: (1) professional society, (2) clinical practices, and (3) general health information websites. “Professional society” was defined as an organization that wants to promote a certain job or interest. “Clinical practice” was defined as a health institution, genetic laboratory, imaging center, hospital, or private practice that provides medical or surgical treatment mainly to outpatients. “General health information website” was defined as the websites from non-clinical institutions that provide general public health information.

Every meaningful text regarding HHI was copied to a separate Microsoft Word (version 2010; Microsoft, Redmond, WA) document. Texts that were not related to education such as webpage navigations, copyright notices, disclaimers, writer information, feedback questionnaires, links, website URLs, references, figures, tables, footnotes, addresses, and telephone numbers were deleted in order not to influence readability scores.

Readability scores were automatically calculated by transferring the texts to https://www.webfx.com/tools/read-able/ (33). Mean, standard deviation, median, minimum, maximum value, frequency, and percentage were used for descriptive statistics. The distribution of variables was checked with the Kolmogorov-Smirnov test. The Kruskal-Wallis was used for the comparison of quantitative data. SPSS 28.0 was used for statistical analyses.

## 3. Results

After applying our inclusion and exclusion criteria, and deleting copies, we investigated 29 websites. We included 4 PEM manuscripts from professional societies, 11 PEM manuscripts from clinical practice websites, and 14 PEM manuscripts from general information websites. Titles and hyperlinks for the 29 included websites are listed in Table 1.

**Table 1:** Titles and hyperlinks for the 29 included professional society, clinical practices and general health information websites websites.

**Professional society**
1. www.enthealth.org
2. www.audiology.org
3. https.medlineplus.gov
4. www.bmc.org
**Clinical Practices**
1. umiamihealth.org
2. https.www.ent.uci.edu
3. www.boystownhospital.org
4. https.www.mottchildren.org
5. blueprintgenetics.com
6. www.sickkids.ca
7. www.pennmedicine.org
8. www.institutimagine.org
9. bredagenetics.com
10. www.schn.health.nsw.gov.au
11. morl.lab.uiowa.edu
**General health information**
1. https.www.hear-it.org
2. aussiedeafkids.org.au
3. https.www.cdc.gov
4. www.cincinnatichildrens.org
5. dizziness-and-balance.com
6. www.verywellhealth.com
7. rnid.org.uk
8. www.babyhearing.org
9. en.wikipedia.org
10. http.www.nationalhearingtest.org
11. www.preventiongenetics.com
12. www.hearingloss.org
13. www.carecredit.com
14. www.orpha.net

The readability scores are shown in Table 2, which indicate that on average 12–16 years of education is required to read and understand the websites focused on HHI. All of the websites surpassed the recommended reading level of sixth grade.

**Table 2:** Overall readability scores for the websites.

	Min-Max	Median	Mean. ± sd
Flesch Reading Ease (FRE)	23.3–69.1	44.9	44.1 ± 11.1
Flesch–Kincaid Grade Level (FKGL)	7.2–15.4	11.7	12.1 ± 2.1
Gunning–Fog Index (GFI)	10.2–18.4	15.1	15.1 ± 2.1
SMOG Grading (SMOG)	7.7–13.7	11.2	11.2 ± 1.5
Coleman–Liau Index (CLI)	10.7–17.2	13.6	13.6 ± 1.5
Automated Readability Index (ARI)	7.0–16.2	11.8	12.1 ± 2.4

Reading difficulty was categorized by US Department of Health and Human Services as 6 grade and below, easy; 7th grade–9th grades were classified as average difficulty and those over 9th grade were classified as difficult (34). There were no manuscripts written at the 6th-grade level or below. While two clinical practice websites were at the 8th-grade readability level according to FRE scoring, only one out of eight general health information websites were at the 8th-grade readability level. In the FKGL scoring, two clinical practice websites were at the 8th-grade readability level. Of the general health information websites, two were at the 8th-grade level, and one was at the 7th-grade level. In the GFI scoring, all manuscripts were above the 8th-grade level. In the SMOG scoring, three clinical practice websites were at the 8th-grade level, one general health information website was at the 7th-grade level, and two were at the 8th-grade level. In the CLI scoring, all manuscripts were above the 8th-grade level. In the ARI scoring, two clinical practice websites were at the 8th-grade level, while one general health information website was at the 7th-grade level, two were at the 8th-grade level. All professional websites were more difficult than the 8th-grade level in all scorings. Although general health information websites had higher easy-to-read scores, the difference was not statistically significant (Table 3).

**Table 3:** Comparison of readability scores between professional, general and clinical practice websites.

			Professional		General		Clinical Practice		*p*
Flesch Reading Ease		Mean. ± sd	42.3 ± 6.9		46.9 ± 10.6		41.2 ± 12.8		0.376^K^
		Median	42.1		47.3		41.6		
Flesch Reading Ease	≥17th grade	*n*-%	0	0.0%	0	0.0%	3	27.3%	* *
	13th–16th grade	*n*-%	2	50.0%	5	35.7%	2	18.2%	* *
	10th–12th grade	*n*-%	2	50.0%	8	57.1%	4	36.4%	* *
	8th–9th grade	*n*-%	0	0.0%	1	7.1%	2	18.2%	* *
Flesch–Kincaid Grade Level		Mean. ± sd	12.6 ± 1.6		11.6 ± 2.1		12.6 ± 2.4		0.473^K^
		Median	12.6		11.7		13.1		
Flesch–Kincaid Grade Level	7th grade	*n*-%	0	0.0%	1	7.1%	0	0.0%	* *
	8th–9th grade	*n*-%	0	0.0%	1	7.1%	2	18.2%	* *
	10th–12th grade	*n*-%	2	50.0%	10	71.4%	3	27.3%	* *
	13th–16th grade	*n*-%	2	50.0%	2	14.3%	6	54.5%	* *
Gunning–Fog Index		Mean. ± sd	15.6 ± 1.4		14.7 ± 2.1		15.5 ± 2.3		0.603^K^
		Median	15.3		14.8		16.2		
Gunning–Fog Index	10th–12th grade	*n*-%	0	0.0%	2	14.3%	3	27.3%	* *
	13th–16th grade	*n*-%	3	75.0%	10	71.4%	5	45.5%	* *
	≥17th grade	*n*-%	1	25.0%	2	14.3%	3	27.3%	* *
SMOG Grading		Mean. ± sd	11.7 ± 1.1		10.8 ± 1.5		11.6 ± 1.7		0.445^K^
		Median	11.7		10.9		11.8		
SMOG Grading	7th grade	*n*-%	0	0.0%	1	7.1%	0	0.0%	* *
	8th–9th grade	*n*-%	0	0.0%	2	14.3%	3	27.3%	* *
	10th–12th grade	*n*-%	4	100.0%	9	64.3%	5	45.5%	* *
	13th–16th grade	*n*-%	0	0.0%	2	14.3%	3	27.3%	* *
Coleman–Liau Index		Mean. ± sd	13.7 ± 0.8		13.1 ± 1.3		14.1 ± 1.9		0.193^K^
		Median	13.8		13.1		14.1		
Coleman–Liau Index	10th–12th grade	*n*-%	1	25.0%	5	35.7%	2	18.2%	
	13th–16th grade	*n*-%	3	75.0%	9	64.3%	8	72.7%	
	≥17th grade	*n*-%	0	0.0%	0	0.0%	1	9.1%	
Automated Readability Index		Mean. ± sd	12.8 ± 1.8		11.5 ± 2.2		12.7 ± 2.7		0.447^K^
		Median	12.6		11.7		12.5		
Automated Readability Index	7th grade	*n*-%	0	0.0%	1	7.1%	0	0.0%	* *
	8th–9th grade	*n*-%	0	0.0%	2	14.3%	2	18.2%	* *
	10th–12th grade	*n*-%	2	50.0%	8	57.1%	4	36.4%	* *
	13th–16th grade	*n*-%	2	50.0%	3	21.4%	5	45.5%	* *

^K^ Kruskal Wallis.

## 4. Discussion

It is very important to customize the readability of PEM to a level that is understandable for patient and parents. Just like the use of specific language by clinicians, these texts should be modified so that they are easy for parents to understand. The AMA has stated that to reach that level, manuscripts addressing medical education for patients should be written at a 6th grade reading level.

Almost half of the parents of children with ear-nose-throat problems search for the medical needs of their children on the internet (35). Most of these parents are not familiar with scientific search engines like PubMed and will probably perform the search using the most accessible and most used search engines, such as Google. Therefore, we assessed the readability of the most useful sources for patient education that could be found with Google (36). Manuscripts were analyzed in three groups: professional society, clinical practice and general health information. Most of the related websites were above the 6th-grade level that is recommended by the AMA (37).

Readability scores are not absolute indicators of reader comprehension. They are only predictors. Using fewer words and shorter phrases in a document does not guarantee that the patient will recall or understand more of the information. A patient's comprehension of the content on a website is impacted by a greater number of factors, including, grammar, layout, diagrams, syntax, audio, and videos. Our study did not take these factors into account since they could not be assessed by the readability tools selected. No gold standard metric is available for evaluating a health-related website. In a study on the optimal development of health-related websites, 33 factors were rated as very important. These include, the design of the website and the use of color, an easy login procedure, availability, and the provision of graphical representations of cartoons, pictures, and related information (38). None of these factors were assessed in this study. Marketing strategies involve web design and colors that have been studied and correlated to how long people surf at certain websites. We should keep in mind most evaluations are in English. Readability is difficult to achieve when educational material is translated in from medical jargon to plain English. If, in addition to this, the English is then translated into other languages readability is likely to be further reduced.

The present study has some other limitations. First, the medical terms, “hereditary hearing impairment”, “genetic deafness”, “hereditary hearing loss”, and “sensorineural hearing loss of genetic origin” were used in the initial search. The prevalence of this polysyllabic word in the selected text may have skewed readability scores by overestimating the difficulty. Second, without considering the quality, the first 50 websites, falling within our inclusion criteria, for each term were included. Third, the Google search that was performed for this research may not be representative of the experience of each individual patient. The internet provides access to a great number of search engines. We went with Google since it is by far the most popular search engine and is responsible for around two-thirds of all searches conducted on the internet (39). Fourth, only the sources written in the English language were evaluated. Future studies may be useful to evaluate the readability of online sources written in other languages.

The quality of PEM is also important; however, it is a challenging concept to quantify. The DISCERN and CRAAP tests were used to evaluate the quality of websites (40). But tools used to evaluate quality are more subjective. The potency of readability evaluation tools is their being objective.

In the existing literature, Joo et al. (2021) have shown that 128 PEM on the American Academy of Otolaryngology-Head and Neck Surgery, the Canadian Society of Otolaryngology-Head and Neck Surgery, and Ear, Nose, and Throat United Kingdom websites were also above the readability level recommended in the FRE, FKGL, and SMOG readability tools (18). Another study by Svider et al. (2013) showed that all materials evaluated were written at levels above the recommended guidelines (19). Kevin et al. (2017) assessed 126 online information resources published by the American Academy of Otolaryngology—Head and Neck Surgery Foundation and determined that the average readability level was at 10th grade or higher (20). If we look at other disciplines, we see this same trend. Ashley and Amanda (2017) showed that documents on swallowing disorders were written at the 11th-grade level (21). PEM on parathyroid surgery were analyzed by Patel and colleagues and they showed that documents were written above recommended level (22). Elysia et al. (2021) analyzed 85 internet-based PEM about nasal septoplasty and concluded that the readability was at the 10th-grade level (23). Benjamin and Winslo (2022) evaluated 26 internet-based educational sources about the branchial cleft cyst and found that only 3.8% were written at or below the sixth-grade reading level (24). Similar results were shown in many fields of medicine besides ear-nose-throat. The studies in the field of orthopedics, plastic surgery, ophthalmology, urology, and radiology reported that the analyzed sources did not meet the criteria of the current readability guidelines (41–45).

In our study, the information relating to HHI provided by professional society, clinical practices, and general healthcare websites were above the recommended readability level. As professional websites utilize medical terminology to increase reading rates, this is not surprising. The high level of readability of clinical practice websites may be reasonable due to the medical terms used, although they still provide patient information. However, general websites also have higher readability scores than recommended, similar to professional society and clinical practice websites. All of the FRE, FKGL, GFI, SMOG, CLI, and ARI measures demonstrated similar results. Even the best SMOG scoring of general websites indicates a 10th-grade readability level. Similarly, although the best level for professional society websites was found in the SMOG tool, this level is even above the 11th-grade level. The clinical practice also has the best score in the SMOG tool, and it is at the 11th-grade level. All these scores are far above the recommended level, the 6th-grade level.

The readability scores cannot evaluate how accurate the website is in terms of science. In some studies, authors used the control lists developed by them to evaluate the scientific accuracy of adenotonsillectomy and ear surgery videos on YouTube (46, 47). However, self-developed control lists may not be validated or reliable. Besides, these control lists may be needed to be developed separately for each subject and this may be time-consuming.

## 5. Conclusion

The treatment of HHI depends on early detection and intervention. Therefore, it is of the utmost importance to provide readable and understandable patient and parent education material. Here we conclude that the available web-based sources of educational material need to improve their readability to serve this goal.

## Main points

• No websites provide information for patient and parents on HHI at the recommended level of AMA 6th grade in our study.
• All professional websites were more difficult than the 8th-grade level in all scorings.
• In the CLI scoring, all manuscripts were above the 8th-grade level.
• In the GFI index, all manuscripts were above the 8th-grade level.
• Only, 5 out 29 searched websites were at an 8th grade level.

## Data Availability

The original contributions presented in the study are included in the article, further inquiries can be directed to the corresponding author.
